# Explanatory predictive model for COVID-19 severity risk employing machine learning, shapley addition, and LIME

**DOI:** 10.1038/s41598-023-31542-7

**Published:** 2023-04-04

**Authors:** Mariam Laatifi, Samira Douzi, Hind Ezzine, Chadia El Asry, Abdellah Naya, Abdelaziz Bouklouze, Younes Zaid, Mariam Naciri

**Affiliations:** 1 Laboratory of Biodiversity, Ecology and Genome, Department of Biology, Faculty of Sciences, Mohammed V University, Rabat, Morocco; 2 IPSS Laboratory, Faculty of Medicine and Pharmacy, Mohammed V University, Rabat, Morocco; 3 Public Health International Consultant, Rabat, Morocco; 4 Faculty of Sciences, IPSS Laboratory, Mohammed V University, Rabat, Morocco; 5 Department of Biology, Immunology, and Biodiversity Laboratory, Faculty of Sciences Ain Chock, Hassan II University, Casablanca, Morocco; 6 Laboratory of Pharmacology and Toxicology, Pharmaceutical and Toxicological Analysis Research Team, Faculty of Medicine and Pharmacy, Mohammed V University, Rabat, Morocco; 7 Research Center of Abulcasis, University of Health Sciences, Rabat, Morocco

**Keywords:** Immunology, Computational biology and bioinformatics, Machine learning

## Abstract

The rapid spread of SARS-CoV-2 threatens global public health and impedes the operation of healthcare systems. Several studies have been conducted to confirm SARS-CoV-2 infection and examine its risk factors. To produce more effective treatment options and vaccines, it is still necessary to investigate biomarkers and immune responses in order to gain a deeper understanding of disease pathophysiology. This study aims to determine how cytokines influence the severity of SARS-CoV-2 infection. We measured the plasma levels of 48 cytokines in the blood of 87 participants in the COVID-19 study. Several Classifiers were trained and evaluated using Machine Learning and Deep Learning to complete missing data, generate synthetic data, and fill in any gaps. To examine the relationship between cytokine storm and COVID-19 severity in patients, the Shapley additive explanation (SHAP) and the LIME (Local Interpretable Model-agnostic Explanations) model were applied. Individuals with severe SARS-CoV-2 infection had elevated plasma levels of VEGF-A, MIP-1b, and IL-17. RANTES and TNF were associated with healthy individuals, whereas IL-27, IL-9, IL-12p40, and MCP-3 were associated with non-Severity. These findings suggest that these cytokines may promote the development of novel preventive and therapeutic pathways for disease management. In this study, the use of artificial intelligence is intended to support clinical diagnoses of patients to determine how each cytokine may be responsible for the severity of COVID-19, which could lead to the identification of several cytokines that could aid in treatment decision-making and vaccine development.

## Introduction

Coronavirus disease 2019 (COVID-19) is a global public health emergency with severe consequences for populations, health systems, and the economy; consequently, finding ways to prevent the virus’s spread is imperative. This disease is caused by the severe acute respiratory syndrome coronavirus 2 (SARS-CoV-2), and its symptoms range from mild fatigue to activation of inflammatory factors due to a large number of inflammatory exudates and erythrocytes entering the alveoli, leading to dyspnea, respiratory failure, and possibly death^1–4^.

Cytokines are water-soluble extracellular polypeptides or glycoproteins ranging in size from 8 to 30 kDa; they are produced by multiple cell types at sites of tissue injury and by immune system cells by activating mitogen-activated protein kinases^5^. In biomedicine, cytokines have risen to prominence as diagnostic, prognostic, and therapeutic agents for human diseases. The “cytokine storm” is a systemic inflammatory response to infections and medications; it causes an excessive activation cascade of immune cells and the production of pro-inflammatory cytokines as a result of an unregulated host immunological response to multiple triggers^6,7^ such as infections, malignancies, rheumatic diseases, etc.

The cytokine storm is an inflammatory response to infections and drugs that activates and produces pro-inflammatory cytokines^8,9^. In this context, a number of studies have examined the predictive value of sTREM-1, acetylcholine, fatty acids, lipids, IL-1a, IL-b, TNF, IFN-g, and other mediator biomarkers in patients with COVID-19. These studies suggested that using these biomarkers could improve the timing of clinical and pharmacological interventions in COVID-19 patients^10–12^.

After SARS-CoV-2 attaches to the ACE2 receptor and infects alveolar epithelial cells, specific mechanisms are activated by the secretion of cytokines, resulting in an acute inflammatory response that includes lymphokine (produced by lymphocytes), monokine (produced by monocytes), chemokine (involved in chemotactic activities), and interleukin (produced by leukocyte and acting on other leukocytes), that directly promote the inflammatory process^13^.

Elevated or decreased cytokine levels in affected individuals, particularly critically ill patients, suggest that cytokine storms may play a role in COVID-19 pathophysiology^14,15^. Consequently, the cytokines normally released by the immune system would activate the most diverse arachidonic acid cascades, which produce severity-determining metabolites^16–19^.

Today, there is no cure for severe COVID-19, and few treatments improve clinical outcomes significantly. Even within the scientific community, many individuals are unable to differentiate between infection and inflammation (in this case, the development of the disease) when discussing SARS-CoV-2 infection. However, there are already anti-infection medications^20,21^.

The medical community is evaluating antiviral and immunomodulatory treatments for the disease in the interim. Antiviral and supportive treatments are unquestionably necessary for the treatment of COVID-19 patients, but anti-inflammatory therapy plays a crucial role in the management of COVID-19 patients because of its ability to prevent further harm, organ damage, or organ failure^22^.

Numerous studies have demonstrated the importance of certain treatments, such as dexamethasone, tocilizumab, and Regeneron’s monoclonal antibody combination, in reducing the risk of death in hospitalized patients^20–26^. Remdesivir; Sotrovimab; Baricitinib; Evusheld^®^ (cilgavimab + tixagevimab); Paxlovid^®^ (nirmatadvir + ritonavir); and Molnupiravir^®^ were also administered during the COVID-19 crisis^23,24^. The majority of these drugs are based on a variety of cytokines, including IL-6, TNF, IFN, IL-10, IL-1, IL-6, IL-2, IL-8, IL-10, IL-12, and IL-10^27–39^. However, biomarker and immune response analysis are still necessary to better comprehend the pathogenicity of the disease and develop more effective treatments and vaccines.

In addition, as computer technology has progressed, Machine Learning (ML) has become a valuable tool for resolving problems requiring mapping multiple inputs to a desired output. Applying ML techniques to determine if common patterns of cytokines can be identified in COVID-19 patients has shown tremendous promise in recent studies.

Natural Language Processing (NLP) was used by Rahman et al.^33^ to extract relevant clinical markers from Electronic Health Records (EHRs). These extracted variables are capable of being modeled to reveal an association between infection severity outcomes and these variables.

Ghazavi et al.^34^ use one-way ANOVA and Receiver operating characteristic curve (ROC) to determine the optimal cut-off values of cytokine levels for classifying COVID-19 severity with the highest sensitivity and specificity.

Gao et al.^35^ developed a Nano plasmonic digital immunoassay by combining a Machine Learning-assisted nano plasmonic imaging strategy with a microfluidic immunoassay platform that overcomes significant limitations for cytokine profiling in actual patient samples.

Patterson et al.^36^ used SMOTE to balance the classes in their Dataset and then developed a random forest classifier to identify relevant cytokines for disease onset.

Cabaro et al.^37^, utilized Machine Learning techniques including Linear Discriminant Analysis (LDA), Tree (CART), and neural networks to develop a cytokine profile of patients with mild and severe COVID-19 symptoms.

Liu et al.^38^ used the Mann–Whitney U test and univariate and multivariate logistic regression models to determine the cumulative mortality rate based on the normal range of cytokines in order to examine the effect of COVID-19 on the secretion of cytokines.

Other comparable machine learning-based experiments were discussed in terms of the creation of predictive models that lacked interpretability despite their high performance. In fact, the issue with ML approaches in healthcare applications is their black-box nature^39–42^, in which the process of achieving a particular output is concealed. Incorporating interpretation frameworks could increase the acceptability of an ML technique designed to combat COVID-19 by incorporating interpretation frameworks. The analytical transparency provided by these frameworks exceeds the capabilities of conventional data analysis techniques.

In this paper, we have adopted the SHAP and the LIME explainer. The adoption of these models is a sophisticated method for enhancing the transparency of machine learning (ML) models, as they provide both a global and a local perspective on how each factor influences the final probability associated with the potential development of the pathology.

The practical implications of employing these models can support clinical diagnoses performed on examined patients to determine how each cytokine may be responsible for the possible development of the disease and, therefore, be treated individually, as well as to suggest several cytokines that could aid in treatment decision-making and vaccine development.

Until now, the analytical methods utilized in the study of COVID-19 have always yielded generic results, whereas dissecting the problem and isolating its components could provide clinical operators with useful information.

In this study, the Mice-Forest model was utilized to fill in missing data, followed by the VAE Deep Learning model to generate synthetic data. Then, multiple classifiers were used to forecast COVID-19 outcomes (Healthy, Non-Severe, and Severe). The outputs of the models were then explained globally and locally using SHAP and LIME. The most predictive attributes for each group (Healthy, Non-Severe, and Severe) were identified and ranked based on the interpretation results.

## Basic concepts

### Shapley additive explanation (SHAP)

The Shapley Additive Explanation (SHAP) algorithm is a technique for explaining the predictions of machine learning models. A game-theoretical approach^43^ assigns a value to each input feature to represent its contribution to the prediction while considering all possible feature combinations.

Natural language processing, computer vision, and healthcare have all used the SHAP algorithm. It has been shown that it provides more accurate and comprehensible explanations for complex machine learning model predictions than other techniques^43^.

The SHAP algorithm is as follows:

Given an input [x_1_, x_2_ …., x_p_] with p as the number of features and a trained model f, SHAP approximates f with a simple model g that can explain the contribution of each feature value^43,44^ and thus determine the effect of each feature on the prediction for each possible subset of features. The formula for model g is as follows:

1 $$g\left( z \right) = \varphi_{0} + \sum\nolimits_{i = 1}^{M} {\varphi_{i} z_{i} }$$

[z_1_, z_2_, …; z_p_] is a simplification of the input x, where the value of z is 1, corresponding to the features used in the prediction of the data, while its value is 0 the corresponding feature is not used. ${\varphi }_{i}\in {\mathbb{R}}$ represents the Shapley value of each feature which is a weighted sum of the contributions across all possible subsets, with the weights being proportional to the number of features in each subset.

The ${\varphi }_{i}$ is calculated by the following equation:

2 $$\varphi_{i} \left( {f,x} \right) = \sum\nolimits_{z \subseteq x} {\frac{{\left| z \right|!\left( {p - \left| z \right| - 1} \right)!}}{p!}\left[ {f\left( z \right) - f\left( {z\backslash i} \right)} \right]}$$

SHAP produces a collection of feature weights^44^ that can be utilized to explain the model’s predictions. These weights account for the interaction between features and provide a more precise and nuanced explanation of the model’s behavior. In other words, SHAP computes the Shapley value of each feature as a player in the learned model, a process that must be performed for all possible permutations of features and requires exponential time. Nevertheless, it is known that SHAP can be efficiently calculated for tree-structured models, and since the learning model used in this work is a gradient-boosting tree, the calculation time for the SHAP algorithm can be decreased (Supplementary Table S1).

### Local interpretable model-agnostic explanations (LIME)

LIME is an interpretable machine learning framework used to explain the independent instance predictions of machine learning models^45^. LIME modifies the feature values of a single data sample and then observes the effect on the output. In accordance with this concept, LIME generates a novel dataset comprised of permuted samples and their respective black box model predictions. Among other techniques, the dataset is created by adding noise to continuous features, removing words (for NLP problems), and concealing portions of an image. On this novel dataset, LIME trains an interpretable model (e.g., a linear regression model, a decision tree, etc.) that is weighted by the proximity of the sampled instances to the required instance and conducts tests to determine what happens to the model’s predictions when the data is modified. An explanation is obtained by locally approximating the underlying model with an interpretable model^46^. Local surrogate models with the interpretability requirement can be expressed mathematically as follows:

3 $${\text{Interpretation }}\left( {\text{x}} \right) = {\text{ arg}}\,{\text{ min}}_{{{\text{v}} \in {\text{V}} }} {\text{L}}({\text{u}},{\text{v}},\pi_{{\text{x}}} ) \, + {\text{w}}\left( {\text{v}} \right)$$

We consider an explainable model v (e.g., a decision tree) for the sample x that reduces a loss L (e.g., binary cross entropy) and measure how close the interpretation is to the value anticipated by the initial model u. (e.g., a gradient boosting model). This procedure is carried out while minimizing the model complexity ω(v). Here, V represents the set of realizable explanations, which, in a hypothetical scenario, could be decision tree models. The closeness measures πx defines the extent of the locality surrounding sample x and are considered in the explanation^46^.

We can conclude that The LIME algorithm is implemented as follows:

- Choose a specific instance of the input data for which the model’s prediction is to be explained.
- The selected instance is perturbed by generating a set of slightly modified data points surrounding the original point.
- Predict the output for each perturbed data point using the black-box model and record the corresponding input features.
- Utilize the recorded input features and output values to train a locally interpretable model, such as a linear regression or decision tree, using the recorded input features and output values.
- Explain the prediction of the black-box model on the original data point using the local model.

LIME has been implemented in numerous fields, including natural language processing, computer vision, and healthcare, to explain the predictions of complex machine learning models. It can be used to determine which input features are most influential in determining the model’s output for a particular instance of input data.

### Variational AutoEncoder (VAE)

Autoencoders are neural networks that are trained to minimize the reconstruction error between inputs and outputs^47^. The most prevalent constraint consists of reducing the dimensionality of the hidden layers so that the neural network retains only the most essential features required to reconstruct the input.

Variational autoencoder (VAE) inherits the architecture of autoencoder but imposes additional constraints on the bottleneck, thereby transforming conventional deterministic autoencoder into a potent probabilistic model^48^.

**Figure 1:**
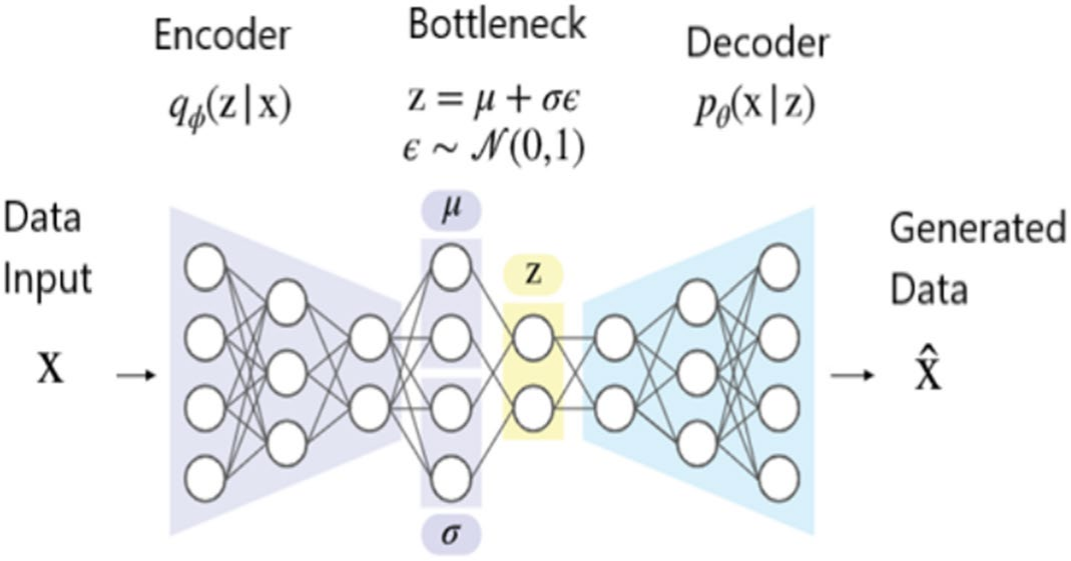
The standard VAE assumes that both X and z follow Gaussian distributions, so the encoder does not output z directly, but rather the distribution parameters of z, i.e. the mean and variance of the Gaussian distribution, and z is reconstructed using reparameterization. The decoder then outputs the mean of the Gaussian distribution with z as the input and fixes the variance.

As shown in Fig. 1, the VAE is composed of an encoder E and a decoder D; the distributions of the encoder and decoder are denoted by q_ϕ_ and p_θ_, respectively. The standard VAE assumes that both X and z adhere to Gaussian distributions, so the encoder does not output z directly, but rather the distribution parameters of z, i.e., the mean and variance of the Gaussian distribution, and z is then reconstructed using reparameterization. The decoder then outputs the mean of the Gaussian distribution with z as the input and sets the variance to a constant value. As the likelihood function of X, this Gaussian distribution is utilized. The model is optimized by calculating the Kullback–Leibler divergence between the prior and posterior distributions of z and the log-likelihood function of X. In conclusion, VAE learns the distribution of z based on X and reconstructs the distribution of X based on z. The following formula expresses the process of encoding and decoding:

4 $$\left\{ {\begin{array}{*{20}l} {\upmu ,\upsigma = E\left( {X,\phi } \right)} \hfill \\ {{\text{z}} = \upmu +\upsigma \upvarepsilon \quad {\text{where}}\, \upvarepsilon \sim {\text{N}}\left( {0,1} \right) } \hfill \\ {\tilde{X} = D\left( {z,\theta } \right)} \hfill \\ \end{array} } \right.$$

where μ and σ are the mean and standard deviation of z, $\widetilde{X }\in {R}^{L}$ is the reconstructed output, ϕ and θ are encoder and decoder parameters.

VAE^49^ is designed to make the distributions learned by the encoder and decoder as similar as possible. Typically, Kullback–Leibler (KL) divergence is used to describe the proximity of two distributions. The objective function of the VAE, therefore, begins with the KL divergence of the two variational distributions:

5 $$\begin{aligned} & {\text{KL }}\left( {q_{\phi } \left( {{\mathbf{z}}\left| {\text{X}} \right.} \right) \, \left\| {p_{\theta } \left( {{\mathbf{z}}\left| {\text{X}} \right.} \right)} \right.} \right) = {\text{ E}}q_{\phi } \left( {{\mathbf{z}}\left| {\text{X}} \right.} \right) \, \left[ {\log q_{\phi } \left( {{\mathbf{z}}\left| {\text{X}} \right.} \right) \, {-} \, \log p_{\theta } \left( {{\text{X}}\left| {\text{z}} \right.} \right) \, - \log p_{\theta } \left( {\text{z}} \right)} \right] \, + \, \log p_{\theta } \left( {\text{X}} \right) \\ & {\text{Equivalent}}\,{\text{ to}}: \\ & \left. {\log p_{\theta } \left( {\text{X}} \right)} \right] - {\text{KL}}\left( {q_{\phi } \left( {{\mathbf{z}}\left| {\text{X}} \right.} \right) \, \left\| {p_{\theta } \left( {{\mathbf{z}}\left| X \right.} \right)} \right.} \right) = - {\text{E}}q_{\phi } \left( {{\mathbf{z}}\left| {\text{X}} \right.} \right)\left[ {\log q_{\phi } \left( {{\mathbf{z}}\left| {\text{X}} \right.} \right) - \log p_{\theta } \left( {{\text{X}}\left| {\text{z}} \right.} \right) - \log p_{\theta } (z)} \right] \\ \end{aligned}$$

VAE’s loss function is expressed as :

6 $$\begin{aligned} L & = - {\text{E}}q_{\phi } \left( {{\mathbf{z}}\left| {\text{X}} \right.} \right)\left[ {\log q_{\phi } \left( {{\mathbf{z}}\left| {\text{X}} \right.} \right) - \log p_{\theta } \left( {{\text{X}}\left| {\mathbf{z}} \right.} \right) - \log p_{\theta } \left( z \right)} \right] \\ & = {\text{KL}}\,\left( {q_{\phi } \left( {{\mathbf{z}}\left| {\text{X}} \right.} \right)\left\| {p_{\theta } ({\mathbf{z}})} \right.} \right) - {\text{E}}q_{\phi } \left( {{\mathbf{z}}\left| {\text{X}} \right.} \right)\left[ {\log p_{\theta } \left( {{\text{X}}\left| {\text{z}} \right.} \right)} \right] \\ \end{aligned}$$

**Figure 2:**
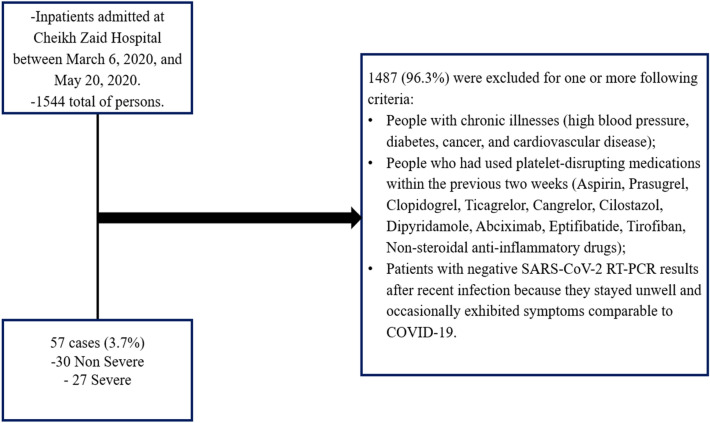
Flowchart of cases recruitment.

The first term is the regularization error of the posterior (|X) and prior p_θ_(z) distributions. Its goal is to minimize the difference between the posterior and prior distributions (Fig. 2).

The second term is the log-likelihood function of X with respect to (|X), p_θ_(X|z ) represents the distribution of X generated by z, and this term computes the difference between X and the reconstructed output $\widetilde{X}$. Consequently, reconstruction error can be written as:

7 $${\text{E}}q_{\phi } \left( {{\mathbf{z}}\left| {\text{X}} \right.} \right)\left[ {\log p_{\theta } \left( {{\text{X}}\left| {\text{z}} \right.} \right)} \right] = \left( {X - \tilde{X}} \right)^{2}$$

VAE successfully combines a variational inference framework and an autoencoder, enabling the model to extract features and generate data more effectively. Several fields, such as image generation, natural language processing, and chemical design, have already demonstrated the potential of VAE^50^.

## Methods and results

**Figure 3:**
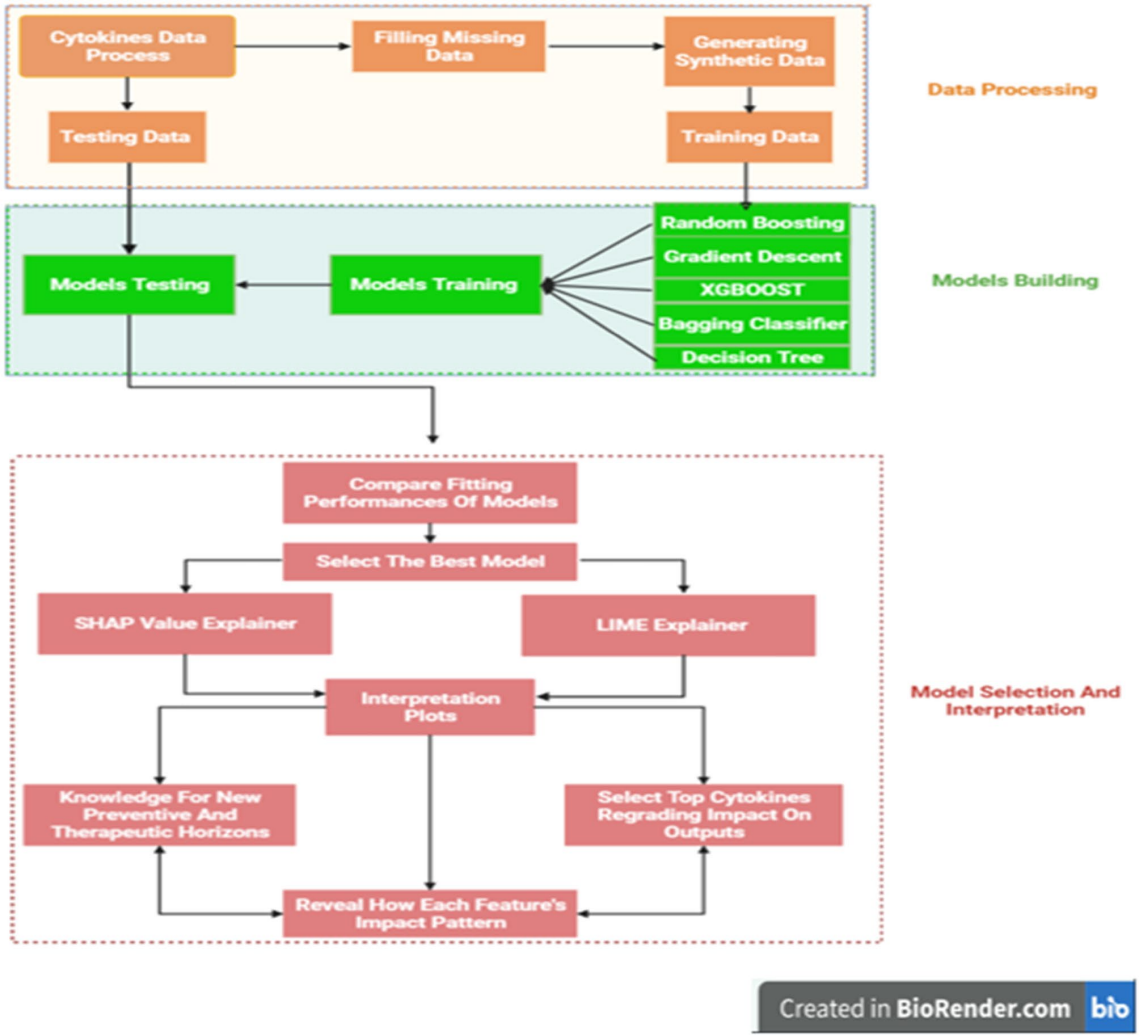
This flowchart depicts the various machine learning models used in this study, beginning with the filling in of missing values, followed by the generation of data, and concluding with the application of predictive models that have proven effective. The best of these models was chosen for interpretation, and LIME and SHAPE were used to interpret the model’s decisions and determine the cytokines that influence the severity of covid 19 disease.

The association between cytokine storm and COVID-19 severity was investigated using advanced machine learning techniques as depicted in Fig. 3 Missing values are filled in after data processing, and synthetic data is generated using the VAE model. Many Classifiers are trained on the synthetic set, and their performance is assessed using real data. Following that, the models’ fitting performances are compared, and the best model is chosen. SHAP and LIME analyses are carried out, and their plots are created to reveal the feature impact in all cases. The top five features of the optimal model are chosen based on the overall attribution values. For all analyses, we used Python 3.6.7. The dataset used and the specifics of how the approaches were used are described in the sections that follow.

*The Recruitment was approved by the Ethics Committee of Cheikh Zaid Hospital (CEFCZ/PR/2020/PR04) and complies with the Declaration of Helsinki. All participants gave their written informed consent and comply with the Declaration of Helsinki.

## Data availability

*The datasets used and/or analyzed during the current study are available from the corresponding author.

### Clinical data

We used a case–control study for our research. All patients were recruited from Rabat’s Cheikh Zaid University Center Hospital. COVID-19 hospitalizations occurred between March 6, 2020, and May 20, 2020, and were screened using clinical features (fever, cough, dyspnea, fatigue, headache, chest pain, and pharyngeal discomfort) and epidemiological histology**.** Any patient admitted to Cheikh Zaid Hospital with a positive PCR-RT for SARS-CoV-2 was considered a COVID-19 case. According to the severity, the cases were divided into two categories: Cases with COVID symptoms and a positive RT-PCR test requiring oxygen therapy are considered severe. Case not requiring oxygen therapy: any case with or without COVID symptoms, normal lung CT with positive RT-PCR. The Controls were selected from Cheikh Zaid Hospital employees (two to three per week) who exhibited no clinical signs of COVID-19 and whose PCR-RT test was negative for the virus. People with chronic illnesses (high blood pressure, diabetes, cancer, and cardiovascular disease) and those who had used platelet-disrupting medications within the previous two weeks (Aspirin, Prasugrel, Clopidogrel, Ticagrelor, Cangrelor, Cilostazol, Dipyridamole, Abciximab, Eptifibatide, Tirofiban, Non-steroidal anti-inflammatory drugs) are excluded from our study (Fig. 2).

Consequently, a total of 87 participants were selected for this study and divided as follows: 57 Patients infected with SARS-CoV-2: Thirty without severe COVID-19 symptoms, twenty-seven with severe symptoms requiring hospitalization, and thirty healthy controls. Table 1 displays patients’ basic demographic and clinical information.

**Table 1:** Patients’ data is presented as mean standard deviation.

Index	Healthy donors	COVID-19 non-severe	COVID-19 severe	p-value		
				Healthy vs non-severe	Healthy vs severe	Non-severe *vs* severe
N° of patients	30	30	27			
Female/Male	15/15	14/16	14/13	–	–	–
Age, years	54.32 ± 9.26	58.12 ± 8.60	61.15 ± 17.82	0.74	0.49	0.96
Weight, kg	87.79 ± 14.53	79.41 ± 16.38	75.68 ± 8.91	0.28	0.37	> 0.99
Platelet number × 109/L	234 ± 63.07	242 ± 56.20	229 ± 37.64	0.56	> 0.99	0.54
ALT, U/L	12.22 ± 4.53	12.47 ± 6.62	19.15 ± 5.08	> 0.99	≤ 0.05	≤ 0.05
AST, U/L	10.95 ± 5.35	17.31 ± 4.26	25.69 ± 6.40	≤ 0.05	≤ 0.05	≤ 0.05
LDH, U/L	325.77 ± 83.46	449.66 ± 83.92	458.50 ± 102.11	≤ 0.05	≤ 0.05	> 0.99
C-reactive protein, mg/L	5.45 ± 3.57	12.16 ± 6.77	19.94 ± 4.88	≤ 0.05	≤ 0.05	≤ 0.05
D-dimers, mg/L	0.36 ± 0.43	0.81 ± 0.48	0.88 ± 0.78	≤ 0.05	≤ 0.05	0.94

ALT stands for alanine aminotransferase; AST stands for aspartate aminotransferase; and LDH stands for lactate dehydrogenase. Unpaired statistical analysis P values were calculated using the student t-test. Statistical significance is defined as p less than 0.05.

The cytokines investigated in our study are displayed in Table 2, it consists of two panels, the first one contains 48 cytokines, while the second panel contains only 21 cytokines.

**Table 2:** Cytokines contained in each panel.

Panel	Number of cytokines	Number of patients	Included cytokines
Panel-1	48	58	RANTES, sCD40L, EGF, Eotaxin, FGF-2, FLT-3L, MIG/CXCL9, IL-1b, Fractalkine, G-CSF, GM-CSF, GROa, IFN-a2, IFNy, IL-1a, PDGF-AB/BB, IL1-ra, IL-2, IL-3, VEGF-A, IL-4, IL-10, IL-5, IL-6, TGFa, IL-7, IL-8, IL-17A, IL-9, IL-12p40, MCP-1, IL-12p70, IL-13, MIP-1a, IL-15, IL-17E/IL-25, IL-17F, IL-18, IL-22, IL-27, IP-10, MCP-3, M-CSF, MDC, TNF, MIP-1b, PDGF-AA,^51–54^
Panel-2	21	29	GM-CSF, IL-8, GROa, IL-3, IFN-a2, IFNy, IL-1a, IL-9, IL-1b, IL-2, IL-4, IL-6, G-CSF, IL-7, IL-10, IL-12p40, IL-5, IL-12p70, IL-13, IL-15, IL1-ra

### Missing data handling process

A data imputation procedure was considered for filling in missing values in entries. In fact, 29 individuals in our dataset had a missingness rate of more than 50 percent for their characteristics (cytokines), therefore our analysis will be significantly impacted by missing values. The most prevalent method for dealing with incomplete information is data imputation prior to classification, which entails estimating and filling in the missing values using known data.

There are a variety of imputation approaches, such as mean, k-nearest neighbors’, regression, Bayesian estimation, etc. In this article, we apply the iterative imputation strategy Multiple imputation using chained equations Forest (Mice-Forest) to handle the issue of missing data. The reason for this decision is to employ an imputation approach that can handle any sort of input data and makes as few assumptions as possible about the data’s structure^55^.the chained equation process is broken down into four core steps which are repeated until optimal results are achieved^56^. The first step involves replacing every missing data with the mean of the observed values for the variable. In the second phase, mean imputations are reset to “missing.” In the third step, the observed values of a variable (such as ‘x’) are regressed on the other variables, with ‘x’ functioning as the dependent variable and the others as the independent variables. As the variables in this investigation are continuous, predictive mean matching (PPM) was applied.

The fourth stage involves replacing the missing data with the regression model’s predictions. This imputed value would subsequently be included alongside observed values for other variables in the independent variables. An Iteration is the recurrence of steps ‘2’ through ‘4’ for each variable with missing values. After one iteration, all missing values are replaced by regression predictions based on observed data. In the present study, we examined the results of 10 iterations.

**Figure 4:**
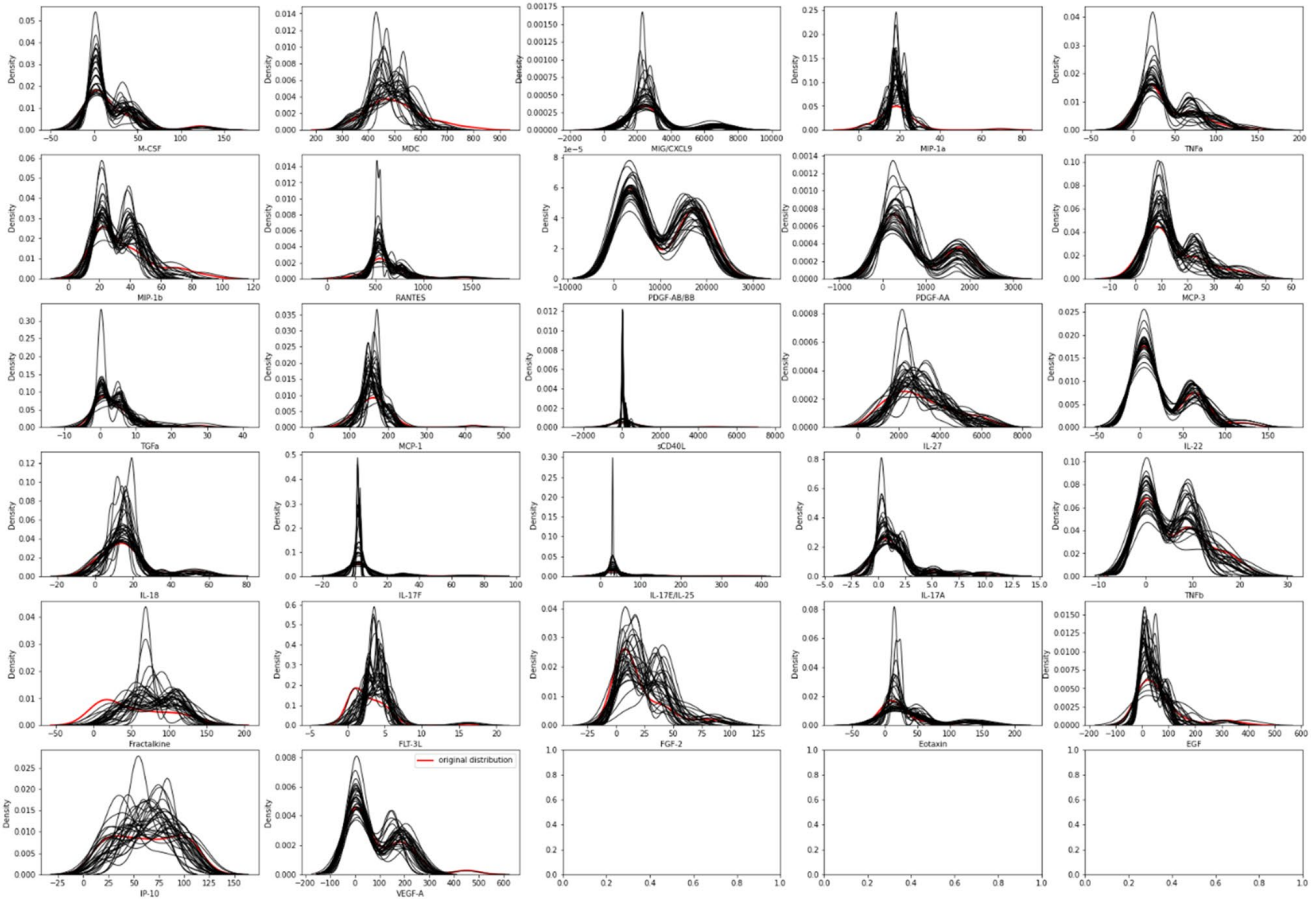
The distribution of each imputation compared to the original data (in red).

The convergence of the regression coefficients is ideally the product of numerous iterations. After each iteration, the imputed values are replaced, and the number of iterations may vary. In the present study, we investigated the outcomes of 10 iterations. This is a single "imputation." Multiple imputations are performed by holding the observed values of all variables constant and just modifying the missing values to their appropriate imputation predictions. Depending on the number of imputations, this leads to the development of multiply imputed datasets (30, in this study). The number of imputations depends on the values that are missing. The selection of 30 imputations was based on the White et al.^57^ publication. The fraction of missing data was around 30%. We utilized the version 5.4.0 of the miceforest Python library to impute missing data. The values of the experiment’s hyper-parameters for the Mice-Forest technique are listed in Table 3, and Fig. 4 illustrates the distribution of each imputation comparing to original data (in red).

**Table 3:** Parameters of mice-forest.

Techniques	Hyper-parameters
Mice-Forest	iterations = 10, imputation = 10 estimators = 50

### Synthetic data generation

Machine learning frameworks have demonstrated their ability to deal with complex data structures, producing impressive results in a variety of fields, including health care. However, a large amount of data is required to train these models^58^. This is particularly challenging in this study because available datasets are limited (87 records and 48 attributes) due to acquisition accessibility and costs, such limited data cannot be used to analyze and develop models.

To solve this problem, Synthetic Data Generation (SDG) is one of the most promising approaches (SDG) and it opens up many opportunities for collaborative research, such as building prediction models and identifying patterns.

Synthetic Data is artificial data generated by a model trained or built to imitate the distributions (i.e., shape and variance) and structure (i.e., correlations among the variables) of actual data^59,60^. It has been studied for several modalities within healthcare, including biological signals^61^, medical pictures^62^, and electronic health records (EHR)^63^.

In this paper, a VAE network-based approach is suggested to generate 500 samples of synthetic cytokine data from real data. VAE’s process consists of providing labeled sample data (X) to the Encoder, which captures the distribution of the deep feature (z), and the Decoder, which generates data from the deep feature (z) (Fig. 1).

**Figure 5:**
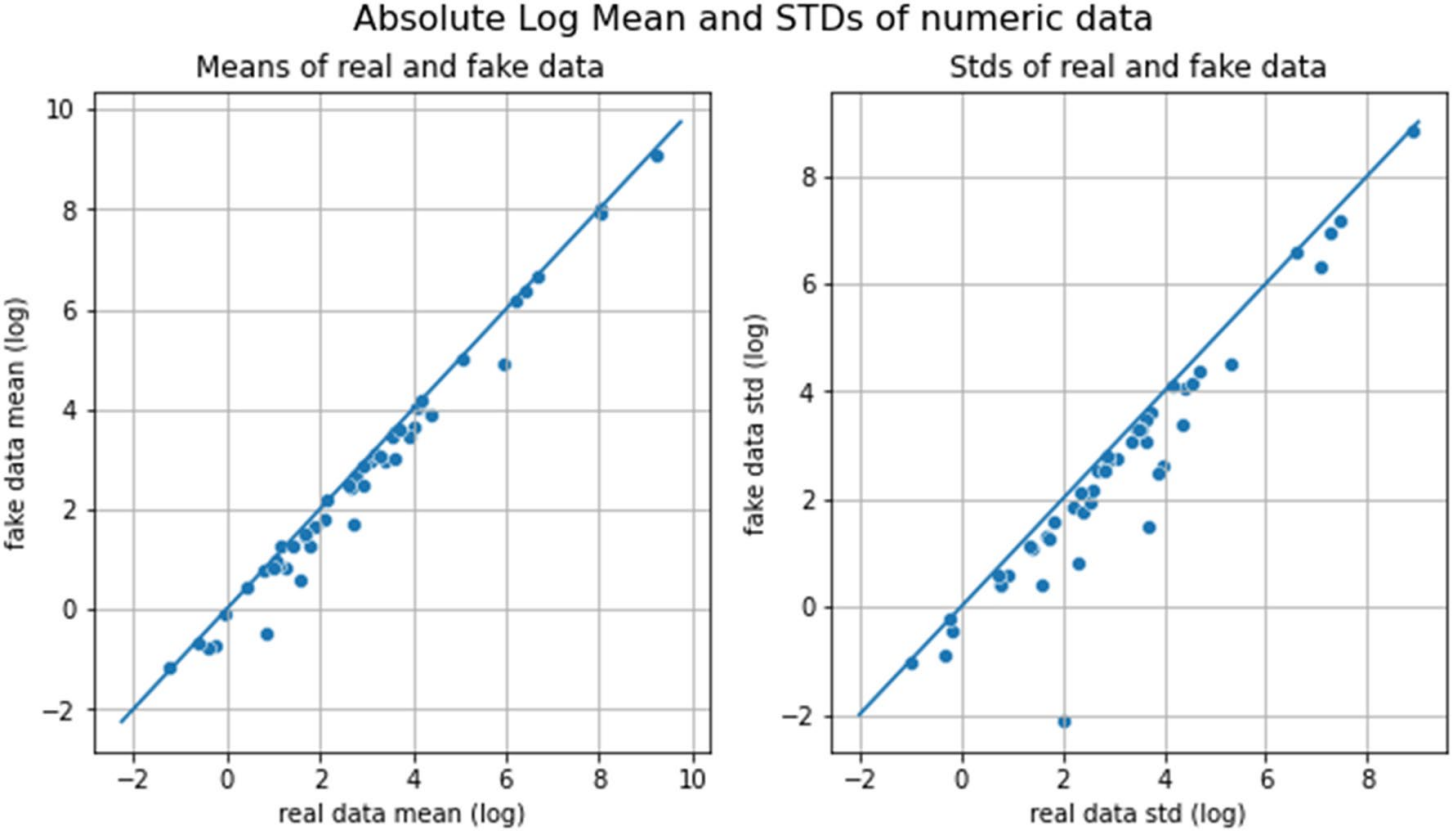
Each point represents a column mean in the real and synthetic data. A perfect match would be indicated by all the points lying on the line y = x.

The VAE architecture preserved each sample’s probability and matched the column means to the actual data. Figure 5 depicts this by plotting the mean of the real data column on the X-axis and the mean of the synthetic data column on the Y-axis.

**Figure 6:**
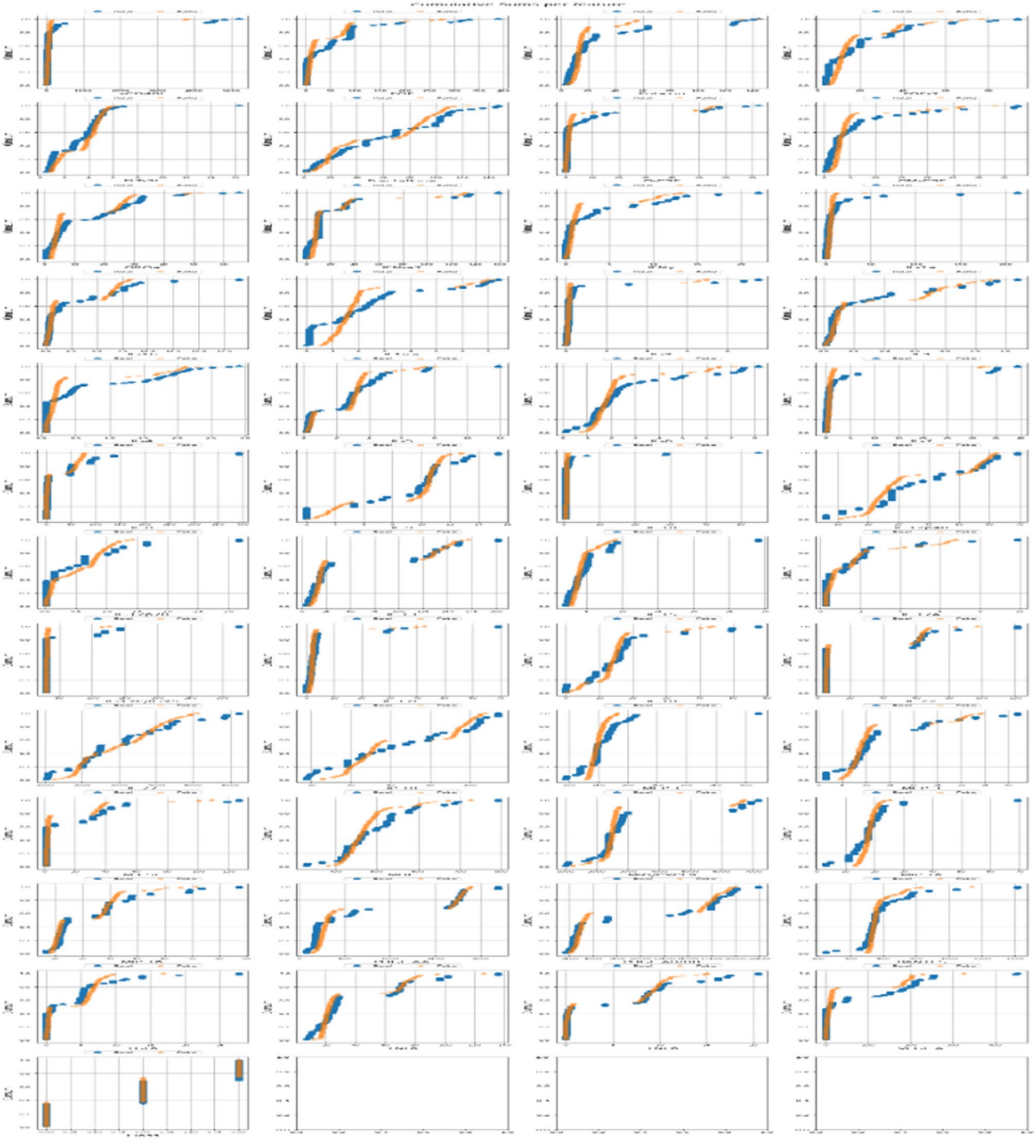
Plots of each feature in our actual dataset demonstrate the similarity between the synthesized and actual datasets.

The cumulative feature sum is an extra technique for comparing synthetic and real data. The feature sum can be considered as the sum of patient diagnosis’ values. As shown in Fig. 6, a comparison of the global distribution of feature sums reveals a significant similarity between the data distributions of synthetic and real data.

### Classification

Five distinct models are trained on synthetic data (Random Forest, XGBoost, Bagging Classifier, Decision Tree, and Gradient boosting Classifier). Real data is used for testing, and three metrics were applied to quantify the performance of fitting: precision, recall, F1 score, and confusion matrix.

**Figure 7:**
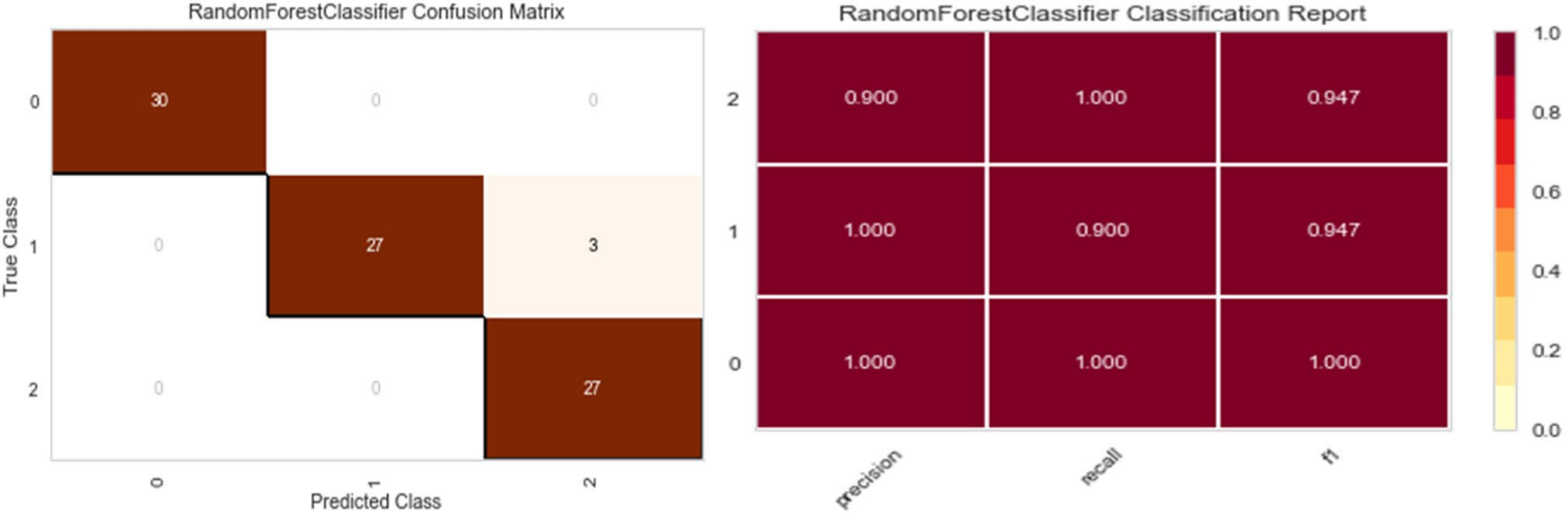
Matrix confusion and Report Classification of Random Forest.

**Figure 8:**
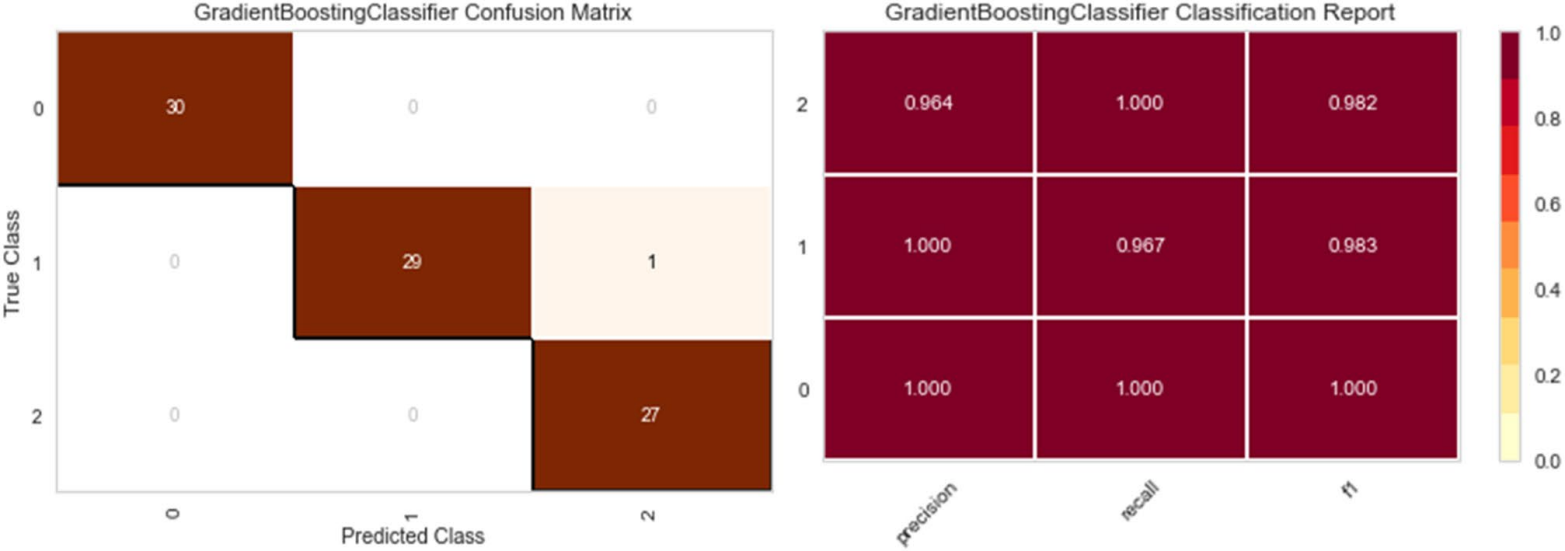
Matrix confusion and Report Classification of Gradient Boosting.

**Figure 9:**
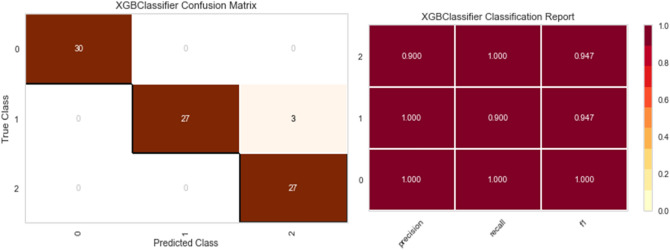
Matrix confusion and Report Classification of XGB Classifier.

**Figure 10:**
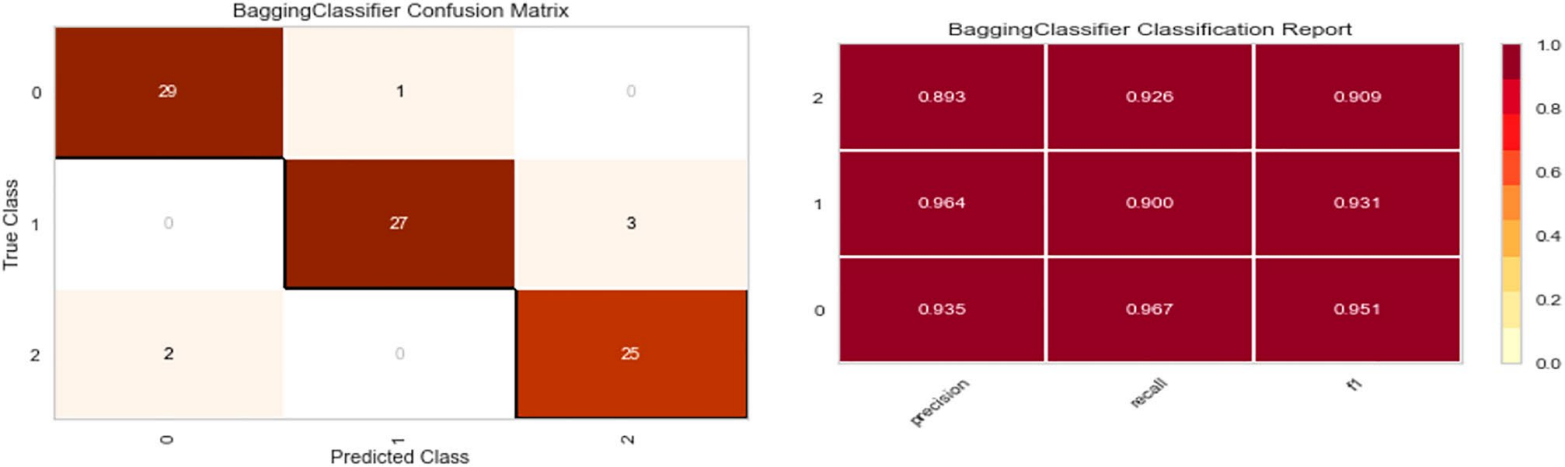
Matrix confusion and Report Classification of Bagging Classifier.

**Figure 11:**
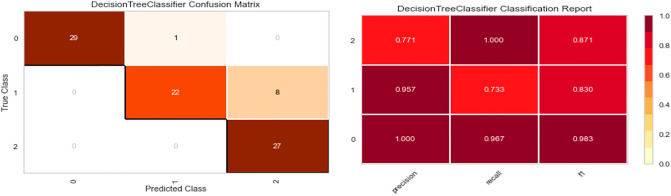
Matrix confusion and Report Classification of Decision Tree.

As shown in Figs. 7, 8, 9, 10 and 11 the performance of the Gradient Boosting Classifier proved to be superior to that of other models, with higher Precision, Recall, and F1 score for each class, and a single misclassification. Consequently, we expect that SHAP and LIME’s interpretation of the Gradient Boosting model for the testing set will reflect accurate and exhaustive information for the cytokines data set.

### Explanations with LIME and SHAPE models

**Figure 12:**
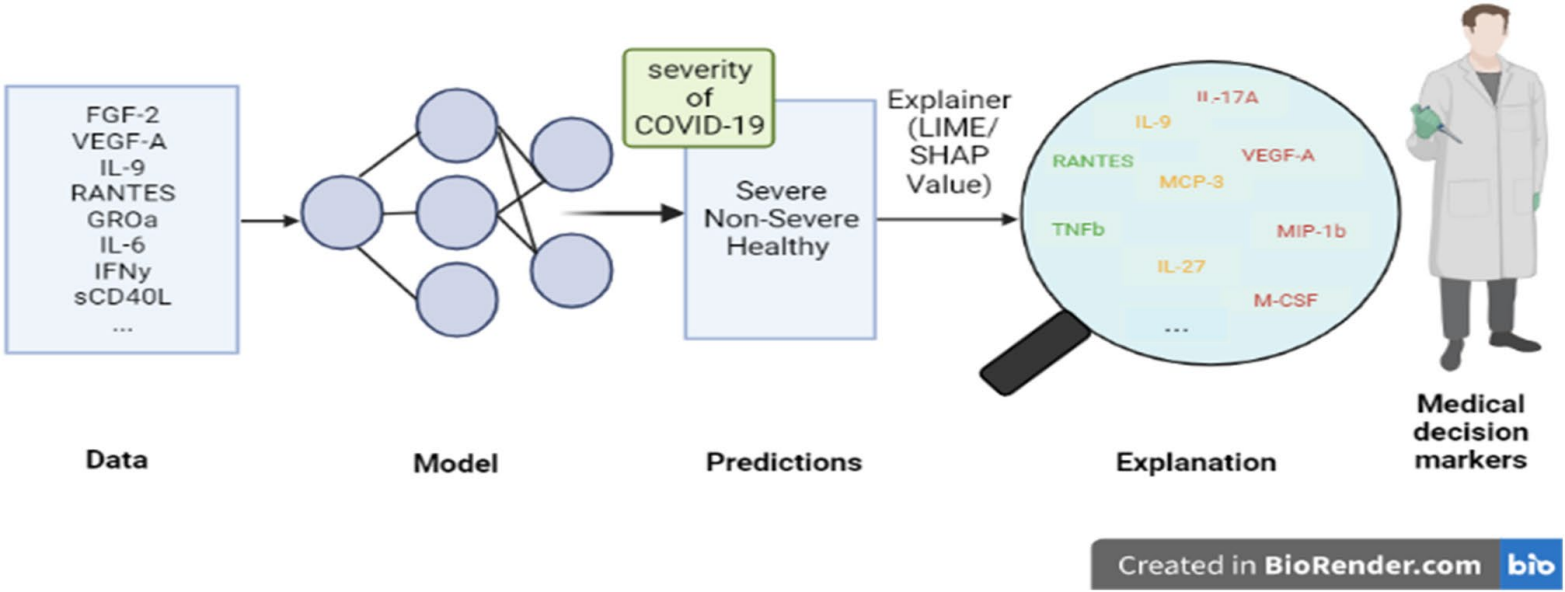
The Flow chart demonstrates how Machine learning can be used to make medical decisions. We entered cytokine data from severe, non-severe, and healthy patients, trained predictive models on cytokine data, and then used LIME and SHAP to explain the most important cytokine for each class of patients (Fig. 12).

Explaining a prediction refers to the presentation of written or visual artifacts that enable qualitative knowledge of the relationship between the instance’s components and the model’s prediction. We suggest that if the explanations are accurate and understandable, explaining predictions is an essential component of convincing humans to trust and use machine learning effectively^43^. Figure 12 depicts the process of explaining individual predictions using LIME and SHAP as approaches that resemble the classifier’s black box to explain individual predictions. When explanations are provided, a doctor is clearly in a much better position to decide using a model. Gradient Boosting predicts whether a patient has an acute case of COVID-19 in our study, whereas LIME and SHAP highlight the cytokines that contributed to this prediction.

#### Explanation of SHAP model

The SHAP explanation utilized in this study is the Kernel Explainer, a model-agnostic approach that produces a weighted linear regression depending on the data, predictions, and model^64^. It examines the contribution of a feature by evaluating the model output if the feature is removed from the input for various (theoretically all) combinations of features. The Kernel Explainer makes use of a backdrop dataset to demonstrate how missing inputs are defined, i.e., how a missing feature is approximated during the toggling process.

SHAP computes the impact of each characteristic on the learned system’s predictions. Using gradient descent, SHAP values are created for a single prediction (local explanations) and multiple samples (resulting in global explanations).

**Figure 13:**
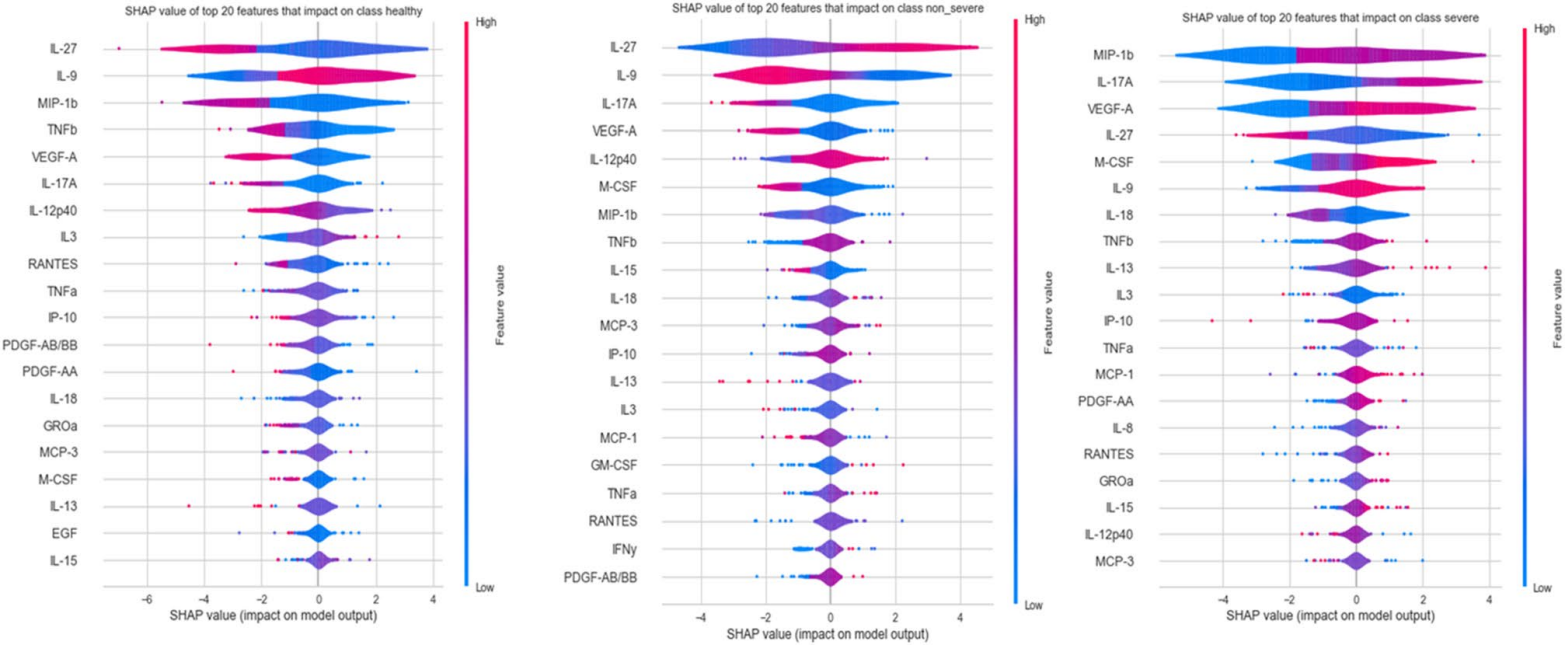
Examples of SHAP values computed for individuals’ predictions (local explanations) for Healthy, Non-Sever, and Sever patients.

Figure 13 illustrates the top 20 SHAP value features for each class in the cytokine data prediction model (Healthy, Severe, and Non-Severe classes). The distribution of SHAP values for each feature is illustrated using a violin diagram. Here, the displayed characteristics are ordered by their highest SHAP value. The horizontal axis represents the SHAP value. The bigger the positive SHAP value, the greater the positive effect of the feature, and vice versa. The color represents the magnitude of a characteristic value. The color shifts from red to blue as the feature’s value increases and decreases. For example, Mip-1b in Figure 8, the positive SHAP value increases as the value of the feature increases. This may be interpreted as the probability of a patient developing COVID-19, severity increasing as MIP-1b levels rise.

**Figure 14:**
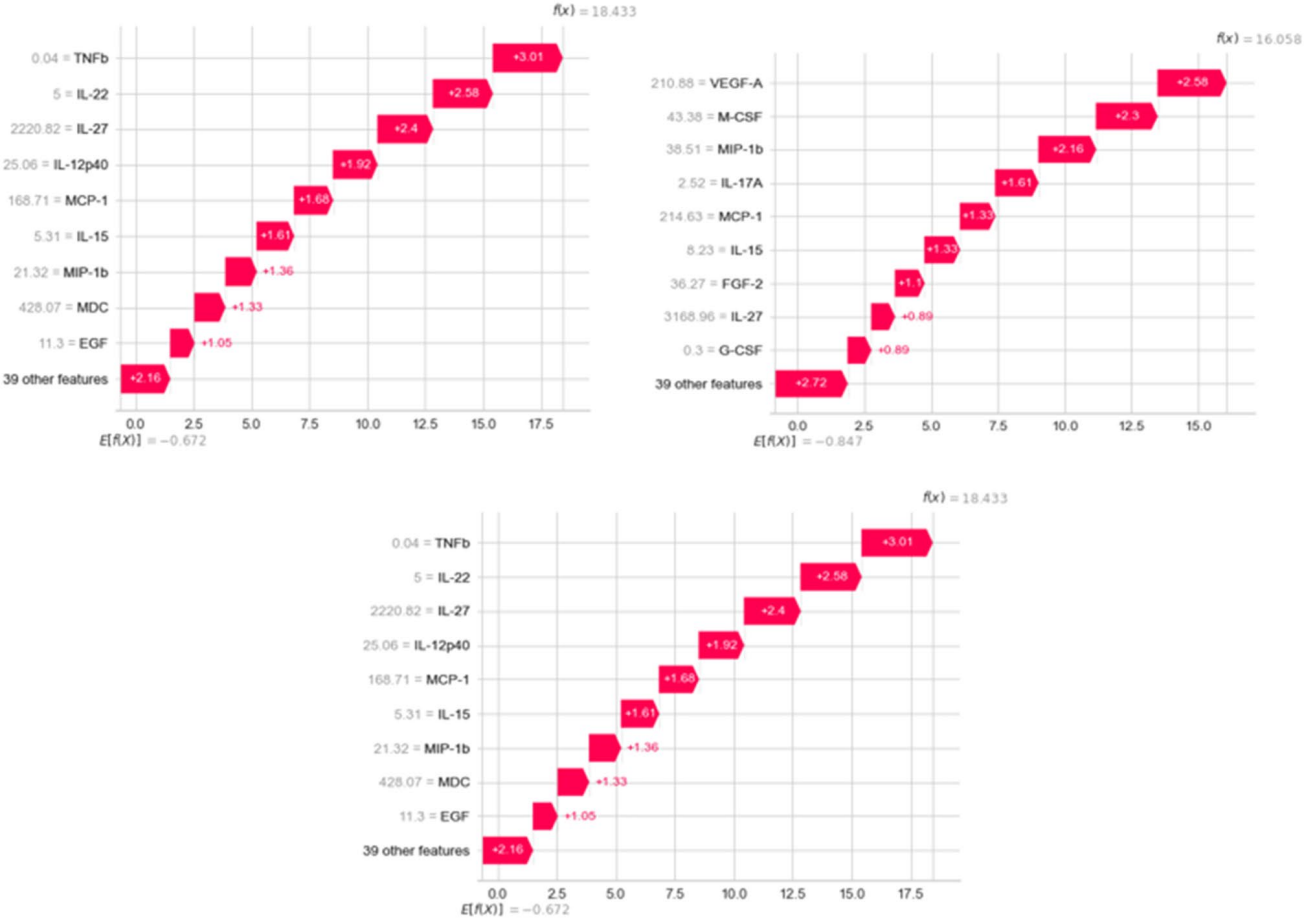
SHAP diagrams of characteristics with varying conditions: Healthy, Severe, and Non-Severe, respectively.

In the situation of a healthy patient, TNF, IL-22, and IL-27 are the most influential cytokines, as shown in Fig. 14’s first SHAP diagram (from left). The second diagram is for a patient with severity, and we can observe that the VEGF-A cytokine’s value is given greater weight. This can be viewed as an indication that the patient got a serious COVID-19 infection due to the increase in this cytokine.

The last SHAP diagram depicts an instance of a non-Severe patient, and we can see that the higher the feature value, the more positive the direction of IL-27. On the other hand, MDC, PDGF-AB/BB, and VEGF-A cytokines have a deleterious effect. The levels of MDC and PDGF-AB/BB cytokines suggest that the patient may be recovering, however, the presence of VEGF-A suggests that the patient may develop a severe case of COVID-19, despite being underweight.

#### Explanation of the LIME model

**Figure 15:**
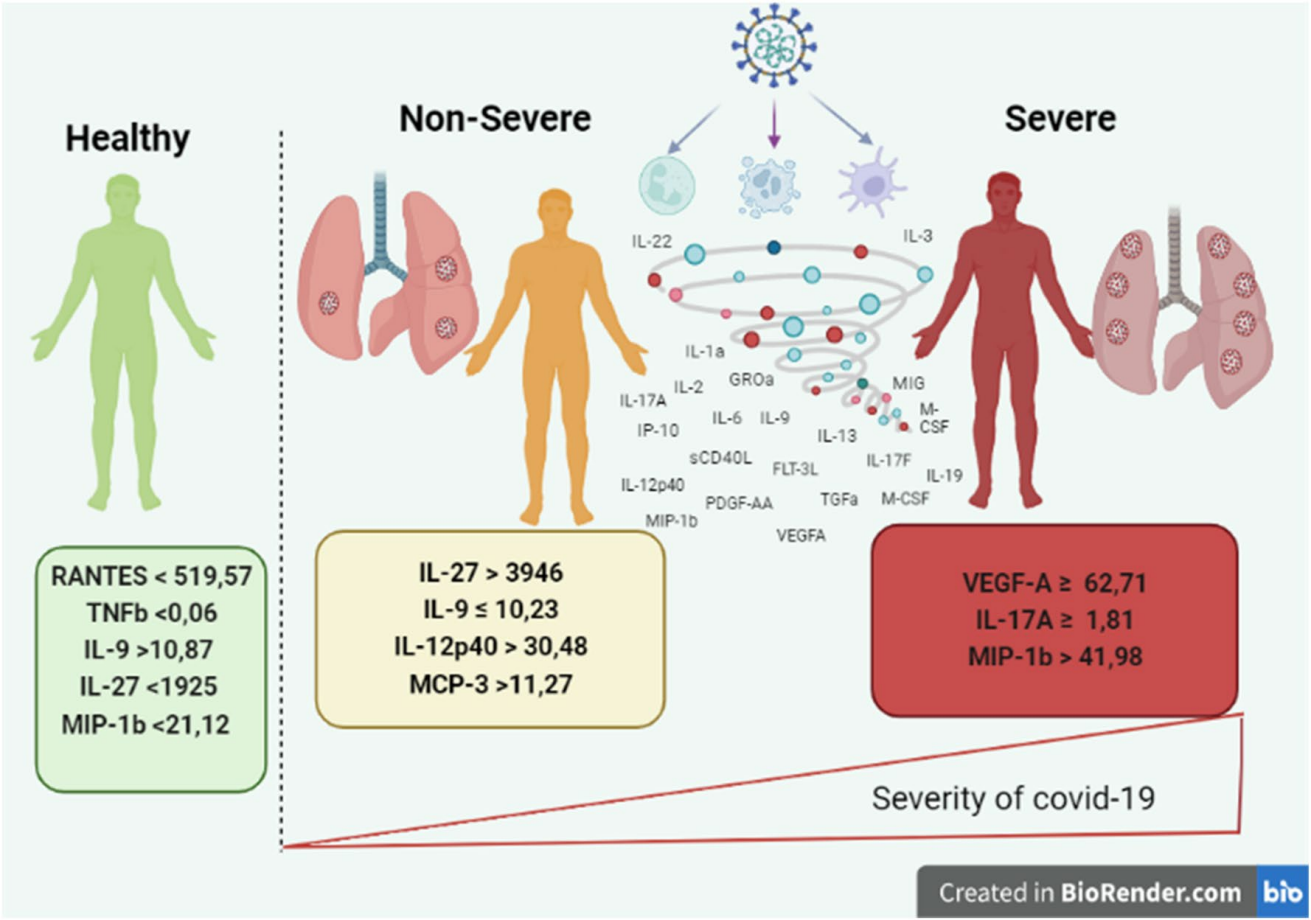
Different Cytokine Profiles Associated with the Progression of COVID-19 Severity.

LIME is a graphical approach that helps explain specific predictions. It can be applied to any supervised regression or classification model, as its name suggests. Behind the operation of LIME is the premise that every complex model is linear on a local scale and that it is possible to fit a simple model to a single observation that mimics the behavior of the global model at that locality. LIME operates in our context by sampling the data surrounding a prediction and training a simple interpretable model to approximate the black box of the Gradient Boosting model. The interpretable model is used to explain the predictions of the black-box model in a local region surrounding the prediction by generating explanations regarding the contributions of the features to these predictions. As shown in Fig. 15, a bar chart depicts the distribution of LIME values for each feature, indicating the relative importance of each cytokine for predicting Severity in each instance. The order of shown features corresponds to their LIME value.

**Figure 16:**
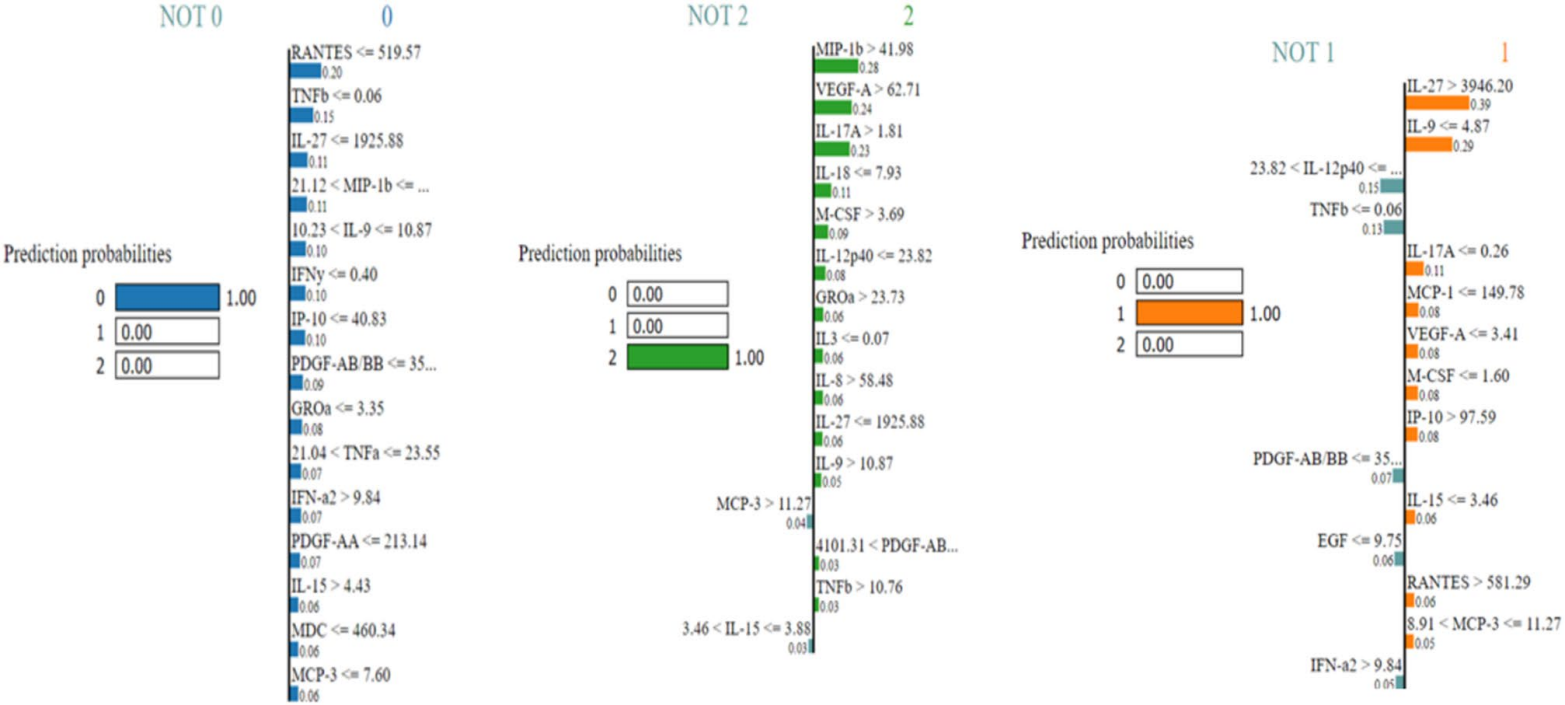
Explaining individual predictions of Gradient descent classifier by LIME.

In the illustrations explaining various LIME predictions presented in Fig. 16. We note that the model has a high degree of confidence that the condition of these patients is Severe, Non-Severe, or Healthy. In the graph where the predicted value is 2, indicating that the expected scenario for this patient is Severe (which is right), we can see for this patient that Mip-1b level greater than 41 and VEGF-A level greater than 62 have the greatest influence on severity, increasing it. However, MCP-3 and IL-15 cytokines have a negligible effect in the other direction.

Alternatively, there are numerous cytokines with significant levels that influence non-Severity. For example, IL-27 and IL-9, as shown in the middle graph in Fig. 14. and that IL-12p40 below a certain value may have the opposite effect on model decision-making. RANTES levels less than 519, on the other hand, indicate that the patient is healthy, as shown in Fig. 16.

By comparing the individual’s explanation of SHAP values to the individual’s explanation of LIME values for the same patients, we may be able to determine how these two models differ in explaining the Severity results of the Gradient descent model. As a result, we can validate and gain insight into the impact of the most significant factors. To do so, we begin by calculating the frequency of the top ten features among all patients for each Explainer. We only consider features that appear in the top three positions, as we believe this signifies the feature’s high value, and we only consider the highest-scoring features that appear at least ten times across all SHAP or LIME explanations (Tables 4, 5, and 6).

Table 4 demonstrates that MIP-1b, VEGF-A, and IL-17A have Unanimous Importance according to the SHAP Value and LIME. In addition, we can remark that M-CSF is necessary for LIME but is ranks poor.

**Table 4:** The top selected feature in the case of Severity for both explainers.

Cytokine	Appearance	
	SHAP value	LIME
MIP-1b	23	14
VEGF-A	24	27
IL-17A	23	23
M-CSF	9	15
IL-9	9	0
IL-12p40	4	1
IL-18	8	4
IL-8	2	0

In the instance of non-Severity, Table 5 reveals that IL-27 and IL-9 are essential in both explanatory models for understanding non-Severity in patients. We can see that IL-12p40 and MCP-3 are also essential for LIME and are highly ranked; hence, we add these two characteristics to the list of vital features for the non-Severity instance. RANTES, TNF, IL-9, IL-27, and MIP-1b are the most significant elements in the Healthy scenario, according to Table 6.

**Table 5:** The top selected feature in the case of non-Severity for both explainers.

Cytokine	Appearance	
	SHAP value	LIME
IL-27	23	24
IL-9	27	22
IL-17A	9	12
VEGF-A	8	3
IL-12p40	9	23
MIP-1b	7	2
IP-10	5	6
MCP-3	6	16

**Table 6:** The top selected feature in the case of Healthy for both explainers.

Cytokine	Appearance	
	SHAP value	LIME
RANTES	6	21
TNF	7	23
IL-9	21	18
IL-27	26	22
MIP-1b	22	16
IP-10	8	7

The elements that explain the severity of the COVID-19 sickness are summarized in Table 7.

**Table 7:** The most significant features selected by SHAP Value and LIME.

Selected cytokines		
Severity	Non-Severity	Healthy
MIP-1b	IL-27	RANTES
VEGF-A	IL-9	TNF
IL-17A	IL-12p40	IL-9
–	MCP-3	IL-27
–	–	MIP-1b

## Discussion

The severity-defining cytokines are VEGF-A, IL-17A, and MIP-1b, as shown in Table 6 and Figs. 13, 14. The non-Severity was linked to IL-27, IL-9, IL-12p40, and MCP-3, and RANTES, TNF, IL-9, IL-27, and MIP-1b were correlated with healthy cases. In addition, the levels of these cytokines are identified based on thresholds detected in the plasma of patients; in the case of Severity, the VEGF-A concentration was found to be greater than^65,66^. VEGF-A, which is essential for vascular endothelial homeostasis, is present in numerous cells and tissues. According to Zhang et al.^67^, VEGF-A plays an essential role in the activation of endothelial cells by binding to cell surface receptors, and the integrity of the endothelial barrier in lung tissue is essential for the regulation of alveolar immune function. In COVID-19, severe lung inflammation and associated immune responses induce apoptosis of epithelial and endothelial cells, which augments VEGF-A production and worsens edema and immune cell extravasation^67^. VEGF-A effects on vascular permeability and neo-angiogenesis^68,69^ is responsible for this factor’s pathogenic properties. Anti-VEGF-A therapy has the potential to be a miracle cure for reducing the severity of the disease in patients.

Researchers have linked COVID-19-related lung inflammation to increased plasma levels of a variety of proinflammatory cytokines, including IL-17A^70,71^. These results are similar with our own, where we discovered higher IL-17A levels in the peripheral blood of infected patients (Table 6 and Figs. 14, 16). As IL-17A promotes the production of other pro-inflammatory cytokines, such as IL-1, IL-6, and TNF, this finding clearly suggests that IL-17A plays an amplifying role in the inflammatory response. Moreover, the increase in IL-17 cytokines seen in these individuals lends credence to the theory that an immunological response leads to severe inflammation^2^. Furthermore, Previous research^72^ indicates that COVID-19-infected patients with severe acute respiratory syndrome had higher levels of circulating IL-17A. According to this idea, the notion of a direct relationship between elevated IL-17A levels and the progression of illness severity becomes more consistent, and our findings indicate that patients develop disease severity when IL-17A concentrations exceed 1.81.

MIP-1b is likewise one of the cytokines with the highest risk when its concentration surpasses 41.98. Multiple disorders, particularly COVID-19, have been shown to exhibit an increase in MIP-1b protein^65,73–75^. The chemokine MIP-1b is a cytokine with the potential to attract monocytes and T cells and may be essential for the recruitment of inflammatory cells to damaged regions^76,77^. Directing inflammatory cells to the airways may result in severe illness or death^77,78^. In addition, MIP-1b was found to be substantial in healthy instances, albeit at a lesser concentration than in severe cases, with MIP-1b levels below 21.12. This result has never previously been reported.

The identification of VEGF-A, MIP-1b, and IL-17A as biomarkers that identify patients with a severe form of SARS-CoV-2 and the determination of their boundaries would enable researchers to more effectively triage and treat patients through the development of therapies and vaccines.

IL-27, IL-9, IL-12p40, and MCP-3 seemed significant in non-Severe samples, and other studies have reported aberrant levels of these cytokines in COVID-19 patients^79–81^.

According to Table 6 and Figs. 14, 16, IL-27 is more relevant in situations that are not severe and exceed 3946. IL-27 is involved in the development of Th1 cells and is a reliable predictive biomarker for COVID-19. On the other hand, we observe that IL-27 levels are low in Healthy cases, which is consistent with research indicating that patients who tested positive for SARS-COV-2 had an immunological imbalance with elevated levels of IL-17A and low levels of IL-27 compared to Healthy sample^82^.

IL-9 is also an indicator cytokine for non-Severity, with a threshold of 10.23 or below. Moreover, the results for the Healthy group indicated that IL-9 appeared significant, but with a higher threshold (IL-9 > 10.87). Consistent with prior research by Ghazavi et al., the concentration of serum IL-9 in COVID-19 patients did not differ significantly from that of the healthy group^34^. In other investigations, cytokine IL-9 levels were shown to be higher in COVID-19 patient groups compared to healthy controls^66,83^, contradicting our findings. The cytokine IL-12p 40 with a threshold greater than 30.48 (IL-12p40 > 30.48) was a crucial bioactive in the non-Severe form. IL-12p 40 is a macrophage chemoattractant that increases the migration of dendritic cells triggered by bacteria. Our results demonstrated that COVID-19-infected individuals express less IL-12p40 than healthy persons, and that IL-12p40 levels decreased as disease severity increased. These results are comparable to those of prior research that showed a decrease in IL-12p40 in intensive care patients^66^.

MCP-3 cytokine is also crucial in mild forms with a threshold greater than 11.27 (MCP-3 > 11.27). MCP-3 was discovered by Yang et al*.*^66^, as an excellent predictor of COVID-19 progression and may serve as a starting point for therapy studies. Non-Severe patients had the highest amount of MCP-3 expression compared to the severe group^84^. Figure 15 summarizes our findings demonstrating the presence of specific biomarkers in healthy samples: RANTES with a threshold of less than 519.57 and TNF with a threshold of less than 0.06. These two biomarkers could be protective factors for these individuals, and research into these biomarkers could be a means for scientists to treat sick people more successfully.

## Conclusion

Cytokines are polypeptide signaling molecules that regulate multiple biological processes via cell surface receptors, such as those involved in adaptive and innate immunity for pro-inflammatory, interleukin, and anti-inflammatory cytokines. Using Machine Learning, we compared the cytokine profiles in this study to determine their significance in the disease’s development. The SHAP and LIME models were used to evaluate the experimental findings regarding the relationship between cytokine storm and the severity of COVID-19 in patients, as well as the influence of various cytokines on severity.

We demonstrated that certain cytokines are likely produced in COVID-19-infected patients and that there are significant increases and decreases in the levels of these cytokines. Significant cytokines were identified as VEGF-A, MIP-1b, IL-17A, M-CSF, IL-27, IL-9, IL12p40, RANTES, and TNF in severe, non-severe, and healthy cases, respectively. These findings suggest that these cytokines may be disease promoters and open new avenues for disease prevention and treatment, this would contribute to a reduction in disease burden in terms of morbidity and mortality. Furthermore, the development of such treatments should consider the cost-benefit ratio, particularly for low-income countries.

## Supplementary Information

Supplementary Table S1.

Supplementary Table S2.

Supplementary Table S3.
